# An easier way to die?—A qualitative interview study on specialist palliative care team members’ views on dying under sedation

**DOI:** 10.1177/02692163251321320

**Published:** 2025-02-21

**Authors:** Jeremias Bazata, Sophie Meesters, Claudia Bozzaro, Violet Handtke, Jan Schildmann, Maria Heckel, Christoph Ostgathe, Claudia Bausewein, Eva Schildmann, For the SedPall Study Group

**Affiliations:** 1 Department of Palliative Medicine, LMU University Hospital, Munich, Germany; 2 Palliative Medicine, Faculty of Medicine, University of Augsburg, Augsburg, Germany; 3 Comprehensive Cancer Center Alliance WERA (CCC WERA), Würzburg, Erlangen, Regensburg, Augsburg, Germany; 4 Institute for Ethics, History and Theory of Medicine, University of Münster, Münster, Germany; 5 Institute for History and Ethics of Medicine, Interdisciplinary Centre for Health Sciences, Medical Faculty, Martin Luther University Halle-Wittenberg, Halle, Germany; 6 Department of Palliative Medicine, Universitätsklinikum Erlangen, Friedrich-Alexander-Universität Erlangen-Nürnberg (FAU), Erlangen, Germany

**Keywords:** Deep sedation, hypnotics and sedatives, palliative care, qualitative research, terminally ill, attitude to death

## Abstract

**Background:**

Professionals’ personal perceptions of sedated patients in the context of palliative care may influence their opinion on sedation as treatment option. However, little is known of palliative care professionals’ perception of patients dying under sedation.

**Aim:**

To explore German specialist palliative care team members’ views on and perception of the dying process under sedation.

**Design:**

Qualitative phenomenological study using semi-structured interviews (*n* = 59). Interviews took place in-person after recruitment via a contact person and were transcribed verbatim. Framework Analysis was used for analysis.

**Setting/participants:**

Physicians, nurses, psychologists, physical therapists, chaplains, and social workers from 10 palliative care units and 7 specialist palliative homecare teams across 12 German cities.

**Results:**

Participants’ views on patients dying under sedation can be grouped into: (i) those who perceived an influence of sedation on the dying process with and without positive and/or negative connotations and (ii) those who saw no difference between dying with or without sedation. Positive connotations referred to the perception of sedation providing an easier path. Concerns were mainly related to the deprivation of patients regarding a conscious dying. The metaphorical description of sedation as “sleep” was common among participants.

**Conclusions:**

The wide range of perceptions of patients dying under sedation may be rooted in different judgements regarding aspects of a good death. Clarifying ideals of a good death with professionals, patients, and relatives before sedation may support transparent decision-making and help avoid conflicts or moral distress.

**What is already known about this topic?**

- Previous studies on sedation at the end of life highlight ethical aspects of sedation, such as the distinction between sedation and physician-assisted dying or challenges in information and decision-making processes.
- Healthcare professionals’ perceptions of sedation influence its application as a treatment option in palliative care, but there is limited research on how healthcare professionals perceive the dying process with sedation.

**What this paper adds?**

- German specialist palliative care team members described their perception of patients dying under sedation, which can be categorized into (i) those who judged sedation as influencing the dying process, both in a positive and/or in a negative way, and (ii) those who did not believe that sedation influenced the dying process.
- Differences in participants’ descriptions on dying with and without sedation seem to echo the discourse around good death.
- This highlights that good death is a concept with differently weighted facets, which can be influenced positively or adversely by sedation.

**Implications for practice, theory, or policy**

- Perception of sedated dying among specialist palliative care team members is heterogeneous, possibly leading to divergent attitudes and approaches to offering and administering sedation at the end of life.
- Clarifying and sharing ideals of a good death with professional teams, patients, and relatives before initiating sedation can ensure transparent decision-making and help avoid conflicts or moral distress arising from differing expectations.

## Introduction

Sedation to relieve intolerable and otherwise intractable suffering in palliative care is a complex and ethically challenging aspect of end of life care. Although there is a large body of work on what exactly constitutes sedation in palliative care, confusion around the definition still exits.^1–3^ Sedation encompasses a large spectrum of reduction of consciousness, ranging from slight drowsiness to coma. Furthermore, sedation can be used intermittently for a predefined period, usually 12–24 h, or as continuous sedation until death, sometimes over the span of multiple days.^4–6^

Many studies exist on the ethical and legal considerations regarding dying under sedation.^7–9^ These considerations include for instance the normative distinction from ending patients’ lives^10–13^ or challenges regarding information and decision-making.^14–16^ Several studies suggest that professionals’ perceptions and opinions of sedation influence their willingness to accept or reject it as a treatment option.^6,17–20^ Consequently, this affects how and when sedation is offered to patients, with regional differences in the evaluation and approach to sedation at the end of life.^6,18,21^ However, there is a scarcity of research on how healthcare professionals perceive the dying process of patients under sedation. Therefore, the aim of this study is to explore German specialist palliative care team members’ views on and perception of the dying process under intentional sedation to relieve suffering.

## Methods

### Design

This qualitative phenomenological study was part of the consortium project “SedPall.”^4^ We followed a broad and inquisitive sociological epistemology, utilizing aspects of action theory, structuralism and post-structuralism in order to grasp our research subject. The SedPall project aimed to develop best practice recommendations on the use of sedative drugs and intentional sedation in specialist palliative care. Semi-structured interviews were conducted to explore a wide range of different perspectives on and experiences with sedation in specialist palliative care. Framework Analysis offers a flexible and pragmatic approach to analyzing large amounts of qualitative data and is not aligned with a particular epistemological or ontological viewpoint. We employed a constructivist epistemological stance and an idealist ontology in accordance with the aim of the study and due to the paucity of qualitative data on sedation practice in Germany. We present this article in accordance with the COREQ checklist (see Supplemental Appendix).^22^

### Setting

Ten palliative care units (hospital wards providing specialist palliative care) and seven specialist palliative home care teams, located across 12 cities in three different federal states of Germany. The involvement of hospital setting as well as homecare setting across different cities in Germany was necessary to inform the development of the national best practice recommendations, which was the overarching aim of the consortium project.

### Population

Physicians, nurses, and other members of the multiprofessional palliative care teams (psychologists, physiotherapists, chaplains, and social workers) participated in the interviews. Inclusion criteria were involvement in at least one case where intentional sedation to relieve suffering was applied in the last year and sufficient German language skills. We took efforts to keep the inclusion criteria as broad as possible to encompass a broad range of experiences and viewpoints.

### Sampling

We employed a maximum variability sampling strategy based on theoretical considerations to ensure representation of perspectives and experiences across all professionals involved in the process of sedation in palliative care. Identified characteristics for sampling included the setting (hospital and homecare), the location of the setting, profession and position, and, as far as possible, age and gender, leading to a predetermined sample size of 50–60 participants.

### Recruitment

We recruited professionals from the participating palliative care units and specialist palliative home care teams. At each recruitment site, one staff member (mostly physicians in leading or management positions) was involved as a “contact person,” who informed the professionals about the study and identified possible candidates. Interested and eligible professionals were then contacted by the researchers (JB and/or VH) by phone or email to schedule appointments and answer remaining questions.

### Data collection

We conducted semi-structured interviews following a topic guide. The topic-guide was discussed with and reviewed by other experienced qualitative researchers at the LMU Hospital and a qualitative expert group at the LMU. The final topic guide comprised five main topics:

- experiences with sedation and understanding of sedation
- indications for and intentions of sedation
- decision-making process and informed consent
- challenges and opportunities
- dying under sedation

The main topics comprised up to seven sub-topics each, which were slightly altered for the respective professional group (physicians, nurses, and other professionals). Regarding the research question, the topic guide included the question whether interviewees perceived a difference in the way sedated patients die compared to non-sedated patients (main topic “dying under sedation.”)

*In your experience, do patients under sedation die differently from others in your daily practice? (Question in topic guide)*

We did not define the concept of sedation in advance but asked the interviewees for their understanding of sedation in palliative care. Moreover, we included the whole spectrum of sedation practices. Therefore, the interview results are not related to one specific form of sedation. Two experienced researchers (VH, JB) piloted the topic guide in six interviews, leading to slight amendments. The same two researchers conducted the interviews at the participants’ workplace between July 2018 and September 2019. During this time, field notes were written and VH and JB discussed preliminary impressions and findings with ES and other qualitative researchers, both from within and outside of the consortium. Data saturation was monitored throughout the analysis process, with no new themes emerging after conducting the planned number of interviews. Additionally, each interviewee was asked to complete an anonymized and standardized form to collect data on sociodemographic and professional backgrounds. The interviews were audio-recorded and transcribed verbatim, including anonymization.

### Data analysis

Data analysis followed the Framework Approach, utilizing MAXQDA ver. 2018.2. After initial readthroughs of part of the interview material (roughly 25%) by two researchers (JB, VH, with support from ES), we developed an initial analytical framework with both inductive (emergent) as well as deductive (thematic) categories retrieved from the topic guide. We then started indexing all interviews, continuously refining the analytical framework during the process (JB, VH, SM, JG). The final version of the analytical framework consisted of 12 categories with 0–10 subcategories each for the whole interview body. Of these 12 categories, 4 categories with 1–10 subcategories each were utilized and interlinked for this research question.

All participant quotes selected for this paper were pseudonymized and translated into English by a professional translator with medical knowledge.

During the whole analysis process, we gathered constant feedback from the research consortium, other experienced researchers from the department as well as from the Patient- and Public Involvement group to ensure the analyses’ robustness. We presented our results at a final conference, where healthcare professionals, including participants, could provide feedback.

### Ethical issues and approvals

All participants provided written informed consent. The study received approval from the Research Ethics Committee of the Medical Faculty of LMU Munich (ref no. 18-191, date April 208).

## Results

We conducted 59 interviews with specialist palliative care team members. The majority was female (69%), had training in palliative care (83%) and worked in the hospital setting (51%), with differences in characteristics between physicians, nurses, and other professions. For a detailed overview of the sample, see Table 1.

**Table 1.** Socio-demographic overview of the interviewees.

Socio-demographic characteristics	Physicians *n* = 26	Nurses *n* = 22	Other professions^c^ *n* = 11	Total *n* = 59
Female, *n* (%)	15 (58)	18 (82)	8 (73)	41 (69)
Work experience in years, median (range)	17 (4-36)	24 (2-40)	23 (1-20)	23 (1-40)
Completed structured training in specialist palliative care, *n* (%)	20 (77)	20 (91)	9 (82)	49 (83)
Intensive care unit/intermediate care experience, *n* (%)	22 (85)	6 (27)	n/a^d^	28 (48)
Setting, *n* (%)	SPC^a^ unit: 11 (42) homecare: 9 (35) other/NR^b^: 6 (23)	SPC ^a^ unit: 12 (55) homecare: 8 (36) other/NR^b^: 2 (9)	SPC ^a^ unit: 7 (64) homecare: 1 (9) other/NR^b^: 3 (27)	SPC^a^ unit: 30 (51) homecare: 18 (31) other/NR^b^: 11 (18)
Medical specialty (completed training), *n* (%)	Internal medicine: 9 (35) Anesthesiology: 7 (27) General Practice: 4 (15) Neurology: 3 (11) Others: 3 (12)			

a Specialist palliative care.

b NR: Not reported; other: For example, chaplains who worked for the whole hospital rather than just one ward.

c Other professions: psychologists, physiotherapists, chaplains, social workers.

d n/a: not applicable; In Germany, the professions included in “other professions” are usually no permanent team members in intensive care units/intermediate care teams and were therefore not included regarding this question.

The accounts of participants who described their perceptions of dying under sedation could be generally categorized into two groups: (i) Those who perceived a difference between dying with and without sedation and (ii) those who did not. Some participants stressed that their perception of differences does not necessarily correspond to the patients’ perception of their own dying process. This made them hesitant regarding assessing the effect sedation has on dying.

*Well, I think that it [dying under sedation] is different, even though we don’t know what a patient feels when they’re dying, right? [. . .] And it is a very peculiar situation. But from the patient’s perspective? I don’t know if for them it’s a better or worse way to die. (Claudia (pseudonym), physician, palliative care unit).*

### Sedation perceived to influence the dying process

#### Alteration of the “natural process” of dying

The majority of interviewees perceived sedation as influencing the dying process.

*Well, the dying process is surely influenced by sedation. I don’t feel like we’re letting everything run its natural course when we use palliative sedation, but instead are influencing the dying process medically. (Caroline, physician, palliative care unit)*

One particularity in this quote is the description of sedation influencing the “natural process” of dying. This distinction between “natural dying” and “sedated dying” was made by many interviewees. Some stated that sedation should be used only if “*the natural dying process became too strenuous*.” Also, participants described dying under sedation as a “premature parting.”

*It’s definitely different, because they die without consciousness. With non-sedated patients who are dying, I can still ask them: What is your feeling? How long are you going to live? Or sometimes they say that it won’t last much longer now. They also say goodbye in a way where everyone involved just knows, there will be no next time. And that’s not the case with sedation. [. . .] It definitely is a premature parting communicatively and I suppose also in terms of consciousness, too. [. . .] I imagine that it is also a premature parting psychologically. (Christel, chaplain, palliative care unit)*

This distinction between “natural” and “less natural” or the perception of sedation as a “premature parting” did not necessarily coincide with negative connotations or perceptions of a patient’s dying process.

#### “An easier path”

Many of the participants who perceived a difference between sedated and non-sedated dying described patients dying under sedation as more “at peace” or “calmer.”

*I can’t generalize here, but often [sedated patients] do die differently. [. . .] And so far, I find sedation more comfortable as an observer. [. . .] More relaxed. [. . .] [Like] sleeping in. (Larissa, nurse, palliative care unit)*

While this observation mainly focuses on the “visible effects” of sedation, others also described a possible effect of allowing the patient to die.

*I guess, I think sometimes it’s an easier path, yes. Sedation often helps [. . .] to let go. And I think [regarding] the [. . .] death throes [. . .], that they don’t need to experience that consciously. (Helena, Nurse, home care team)*

This idea of sedation as an “easier path” was regularly associated with the opinion that dying was often a “struggle” with the patient “fighting” against their death. Sedation was thus seen as helping the patient to “let go.” This is also clarified and expanded upon in the following quote:

*Many of our patients are oncology patients, and they often have been fighting for many years already. And they are fighting until the end. And obviously you can tell them: “You don’t have to fight anymore”, but what they have internalized over time is very difficult to let go of. And if the situation is relieved via these drugs, then we can move on. [. . .]I think it’s easier to let go of this struggle and this will to live and this clinging on then. (Henrietta, Nurse, palliative care unit)*

The interviewees also reported that the patients’ relatives perceived sedated dying as peaceful. With the term “relative”, we include family as well as other close persons.

*With the patients who have died here in our ward, we often hear from the bereaved afterwards, that it’s hard that he [the deceased] is no longer here, but that they were really glad that he could leave without feeling any pain, that he could be under sedation and that he fell asleep peacefully. (Jasmin, psychotherapist, palliative care unit)*

One physician went as far as saying that patients who died under sedation “*relieved the burden on those who stay behind.*”

#### Making the patients “go to sleep”—With positive as well as negative connotations

Many participants also expressed views equating dying under sedation with the image of the patient being peacefully at sleep. This phenomenon was especially common among nurses and chaplains, who often compared this to the (in their eyes) common idea of the “ideal death.”

*I think it’s different for some in so far as it [sedation] makes it easier to cross this threshold. Kind of like how a lot of people wish they could just go to bed in the evening and not wake up the morning after. (Julian, chaplain, palliative care unit)*

In the following quote, one participant also described that this idea of sleeping as “ideal dying” is commonly shared by patients as well as their families and friends:

*Dying is always connected with this concept of falling asleep. . . .] When you’re asleep, you’re fine. You’re relaxed. So, I think that it is already associated with positive thoughts. [. . .] If you ask them, many people will say that they don’t want to wake up anymore or fall asleep. And this gentle falling asleep is what you achieve with sedation at the end of the day, at least to the relatives and patients and this is why [it’s positively received]. You ask them: “What do you want?” – “To fall asleep.” (Caroline, physician, palliative care unit)*

Dying under sedation was also described by participants as an opportunity to “simply slumber one’s own demise.” However, it should be noted that the German word “verschlafen,” which was originally used here, has an ambivalent connotation. It implies peaceful, deep sleep, but it is also commonly used to describe the act of oversleeping and missing out on something.

A minority of participants took a more critical stance toward the sleep metaphor commonly used to describe sedated dying, with some stressing the possible difference between outside observation and patients’ experiences:

*So, if you do it [sedation] too quickly, in the sedated state, I can often notice during working with their bodies, that [. . .] while they are calm on the outside, inside there will still be a lot of restlessness and movement, for example, that perhaps they’re dreaming or still thinking or feeling, but can no longer act accordingly. So sometimes a patient is even more helpless under sedation because it is not calm [on the inside]. [. . .] Even when we sleep, we have dreams, no? Sometimes even nightmares. And maybe you’ve already experienced it that you’re having a nightmare and you just can’t move, can’t get out of it. And those things happen under sedation, too. These things aren’t necessarily gone. The patient is calm on the outside. But something is raging inside, sometimes. [. . .] Some people also say after waking up [. . .] that it was terrible, that they were in hell or something. (Alice, physiotherapist, palliative care unit)*

Thus, “sleep” can have two different, diametrically opposed meanings: sleep can be calm and suggest that the patient is in a state of peaceful rest or sleeping patients can be suffering, without the ability to communicate this or wake up by themselves.

#### Depriving people of a conscious dying and of saying goodbye

The most common aspect raised by interviewees who perceived a negative influence of sedation, was that sedation ended the patient’s experienced life prematurely by rendering them unconscious and unable to communicate, which worried healthcare professionals.

*I think dying in somewhat of an alert state of mind is for me still more dignified, perhaps. I think that is important to a certain extent, that, if that is desired, that he [the patient] is able to perceive his environment, even if perhaps only in a twilight stage. And from my point of view, I think there is a difference in whether I have artificially created this twilight state or whether it happened naturally. [. . .] I believe that [with sedation] many things get lost and perhaps we might also be cutting some life paths or life-endings short sometimes, no? Or the patient’s experiences. And when it comes to these questions, I would like to interfere as little as possible, but instead, give the person the chance to finish their life path on their own. (Yannick, nurse, home care team)*

Yannick here also addressed the idea of “natural dying,” which might be “cut short” by sedation.

A similar view is expressed in the next quote, which additionally stresses that a conscious dying process which includes some degree of suffering is a productive part of personal development.

*I don’t want this to be misunderstood, but [sometimes] through this suffering an opening-up is being made possible. And if I take that away from a person, then I’m taking away something very essential in their journey to dying. It is my experience, that people actually, when they are accompanied and when they go through this suffering [. . .] That they then suddenly reach this meaningful state of mind. Or they come to accept that as a human being, dying is part of the human experience and part of life. [. . .] And that dying is also a part of my spiritual, mental, and of course also physical, but mainly spiritual development. Reaching a point where you are able to accept death. Or you are able to accept this deathly illness. And you take these opportunities away from people if you sedate them too quickly. (Alice, physiotherapist, palliative care unit)*

Some interviewees emphasized that it made a difference whether or not patients could say their goodbyes to family and friends—something that might be “taken away” or influenced by sedation.

*You always have to question how actively the patient can still say goodbye on their own or how natural this goodbye is. If you sedate [with drugs], you overwrite the natural sedation and it becomes a different goodbye. If there’s a clear cut where you switch off the consciousness, then it’s different from a patient becoming sleepier through internal and physical circumstances. Certainly. (Zora, physician, home care team)*

### Sedation perceived not to influence the dying process

Other participants stated that they perceived no universal effect of sedation on a patient’s dying process. These interviewees can be divided into two groups: those who generally thought that sedation did not affect the patient’s dying process and those who stated that dying is an inherently individual experience which can never be generalized.

Among those who thought that sedation did not affect the dying process, a commonly offered explanation was that sedation was simply a medical tool to “help them [the patients] reach their destination,” which is a burden-free death.

*No. There are just those patients that don’t need sedative medication. And it’s always quite, quite natural and beautiful, if it happens that way. Many may still need a little something to relax them but I don’t feel like dying itself is different then. (Antoinette, physician, palliative care unit)*

These arguments were almost exclusively presented by physicians.

The other aforementioned group consisted of few participants who either referred to the fundamental uniqueness of dying (“everyone is dying their own death”) which makes it hard to assess the effect sedation has on the process, or to the fact that sedation does not bring about “guaranteed results” (e.g. tranquility).

*I have seen both. [. . .] I have seen some patients who fell asleep peacefully under sedation. Then there were patients where sedation didn’t really help, where they were still restless, or/ but also, the other way around, patients who were able to go on their way peacefully without sedation. [. . .] Every situation is so unique that you can’t simply say [. . .] sedation automatically has this or that effect. (Isabelle, physician, home care team)*

Some participants also stated that assessing the effect of sedation on the dying process is difficult, since advanced illness tends to cause a natural state of somnolence.

*At some point they all get tired, even if they don’t take anything. They all fall asleep eventually. Some may need a little something, others are fine without and are just so ill that they sleep a lot. I don’t know [if sedation changes anything about the dying process]. No, I don’t think it does. (Quartilla, nurse, homecare team)*

## Discussion

### Main findings

In this qualitative study we identified specialist palliative care team members’ views on dying under sedation. These perceptions can be categorized into two groups: (i) Those who perceived an influence of sedation on the dying process. These participants described an influence on the natural process of dying with the potential to provide an easier path with sedation and the risk of depriving people of a conscious dying. Sleep analogies were often used to talk about sedation. (ii) Those who saw no difference between sedated and non-sedated dying, either because dying is an inherently singular experience that cannot be generalized or because they saw sedation as too unpredictable to ensure certain effects.

The perception of dying with sedation may be closely related to the ideas about what constitutes a good death.^13,23–26^ Therefore, we linked the results with the concept of a good death for data interpretation.

### Dying under sedation and how it is related to what constitutes a good death

The discussion around what constitutes a good death is not new^27–29^ but it is currently gaining relevance in and outside the field of palliative care.^30,31^ The COVID-19 pandemic for example as well as legislation about assisted dying in some countries has contributed to a broader societal awareness about conditions for a good death.^32^ Furthermore, there is a growing recognition of the importance of patient preferences and the need for individualized processes of decision-making,^30,33^ that are often claimed as means to reach a good death.^30,34^

Since health care systems and the societal role of death and dying are culturally highly specific and influenced, and as dying is a very individual process, there is no single concept of a good death.^35^ Different narratives and ideals of a good death have been developed in different medical settings. Some emphasize the importance of specific approaches, like spiritual care,^36^ others explore what a good death can mean in a specific medical context, for instance in anatomy,^37^ or for a specific patient population, like children,^38^ or residents in long term care.^39^ Krikorian et al.^40^ identified six core ideals of a good death: control of pain and symptoms, clear decision-making, feeling of closure, being seen and perceived as a person, preparation for death, and being still able to give something to others. Depending on the value one assigns to each of these aspects, different and even opposing ideals of a good death may result. The answers of our participants’ to how sedation influences their perception of the dying process mirrored the value they give to certain aspects of a good death. Those who described sedation with a positive connotation argued that sedated dying is free from (unnecessary) suffering and thus “better,” or an “easier way to go.” They also stressed the positive value of patient autonomy and decision-making. Some participants also stated that sedation eased the psychological burden of the patient as well as their relatives since it gave them a clear “ending point.” Here, the aspect of feeling of closure becomes evident. Conversely, those who perceived a difference between sedated and non-sedated dying with negative connotations often argued that it deprived patients of the opportunity to die a conscious death, which they valued highly. In accordance with Krikorian et al.’s^40^ descriptions of the core ideals of a good death, this could be interpreted as sedation taking away preparing for death or being seen and perceived as a person. Sometimes, these aspects were explicitly valued higher than (or at least equal to) being pain-free.

This perception of sedation either serving or hindering a good death also influences the way it is being talked about in the interviews: deep sedation was often described as a state of (positively connotated) “sleep” or even “slumber,” while scientifically, it is being described as closer to “unconsciousness” or “comatose.”^9,41^ These expressions might also indicate a cultural shift toward a more medicalized and “clean” view of dying, where the process is seen as controlled and confined to certain spaces and timeframes (e.g. a palliative care unit in the last days before death).^27,32^ Our data emphasize that relieving suffering is not only beneficial for patients, but also for their relatives and healthcare professionals, creating an image of peaceful dying (as opposed to fighting against death and suffering) and closure.^27,42^

These examples demonstrate that different ideals of a good death imply a different evaluation of dying under sedation. A good death is a complex ideal that can consist of different and sometimes diverging aspects that can lead to ambivalences in the evaluation of dying under sedation. While sedation could for example positively influence pain relief, it might have detrimental effects on the social aspects of dying, preventing patients from communicating in their last days. While Krikorian et al.^40^ stressed the lack of a clear definition of what exactly constitutes a good death, and that the core themes differ from person to person, there seems to be a high priority of symptom and pain relief across existing studies,^40,43^ possibly leading to growing acceptance and higher occurrence of rapid sedation to unconsciousness at the end of life.^21,41,44^ Meier et al.^44^ described being pain-free as one of the “top themes” when it comes to defining a good death, with others comprising emotional well-being and preferences for the dying process. Nina Streeck^45^ even described a “tabooization” of suffering in the field of palliative care.

### Strengths and limitations

The main strength of this study is the broad range of perspectives it presents: physicians, nurses, and other members of specialist palliative care teams from different professional as well as demographic backgrounds were included. By recruiting from different palliative care units and specialist palliative home care services, we managed to cover views from different settings. There are also some limitations. As sedation is a highly sensitive topic, there was a risk of social desirability bias during the interviews, which we tried to match by training the interviewers to provide a safe atmosphere and stressing the anonymity of the interviews. During recruitment, the contact persons at the recruitment sites may have inadvertently acted as gatekeepers by suggesting certain interview partners from the specialist palliative care teams without any clear guidelines or reasons for this pre-selection. However, the results cover a wide range of opinions and perspectives, with no indication of setting dependency. We explored the entire range of sedation practices, acknowledging that sedation is often a gradual process. Participants used their own definitions and understandings of sedation. While this approach reflects real-world variability, it also introduces a limitation due to differing interpretations among participants. Lastly, it should be stressed that the ideals of a good death and dying are based on culture and history^28,36^ which limits generalizability of the findings of this study due to the culturally rather homogenous participant group.

### Practice and policy implications

This study provides important insights into the effects of sedation on specialist palliative care team members’ perceptions of a patient’s dying process, which may not only influence their opinion on sedation as a treatment option but also their practice. It highlights that, in the majority of cases, sedation was perceived to influence the dying process and, depending on this perception, was addressed in a certain way. This implies the risk of sedation either being offered too seldom or too late or pre-emptively, depending on the perception of dying under sedation and its relation to views of a good death. Clarifying and sharing ideals of a good death within professional teams and with patients and relatives prior to initiating sedation may therefore help to ensure transparent decision-making about sedation. It may also help to avoid conflicts or moral distress that may arise from differing views of what constitutes a good death. Guideline recommendations and training of professionals in specialist palliative care should address these aspects to further promote best practice of intentional sedation to relieve suffering.

## Supplemental Material

sj-pdf-1-pmj-10.1177_02692163251321320 – Supplemental material for An easier way to die?—A qualitative interview study on specialist palliative care team members’ views on dying under sedation Supplemental material, sj-pdf-1-pmj-10.1177_02692163251321320 for An easier way to die?—A qualitative interview study on specialist palliative care team members’ views on dying under sedation by Jeremias Bazata, Sophie Meesters, Claudia Bozzaro, Violet Handtke, Jan Schildmann, Maria Heckel, Christoph Ostgathe, Claudia Bausewein, Eva Schildmann and For the SedPall Study Group in Palliative Medicine
