# Supplementary material for: An easier way to die?—A qualitative interview study on specialist palliative care team members’ views on dying under sedation
Source: Palliat Med. 2025 Feb 21;39(4):517–26. doi: 10.1177/02692163251321320 (PMC11977801; doi:10.1177/02692163251321320)
Supplement: sj-pdf-1-pmj-10.1177_02692163251321320 – Supplemental material for An easier way to die?—A qualitative interview study on specialist palliative care team members’ views on dying under sedation [file sj-pdf-1-pmj-10.1177_02692163251321320.pdf]

COREQ Reporting Checklist SedPall

Domain 1: Research team and reflexivity	
Personal Characteristics	
1. Interviewer	Detailed in methods section "Data collection" (p3):<br>Interviewer: Violet Handtke (VH), Jeremias Bazata (JB)
2. Credentials	VH: Dr.sci.med., Dipl.-Biol.<br>JB: Master of Arts Sociology<br>Sophie Meesters (SM): Dr. rer. biol. hum., Master of Public Health<br>Eva Schildmann (ES): MD/Dr. med., Master of Science Palliative Medicine<br>Claudia Bausewein (CB): MD/Dr. med., PhD, Master of Science Palliative Medicine<br>Claudia Bozzaro (CBo): Dr. phil, Master of Arts<br>Jan Schildmann (JS): MD/Dr. med., Master of Arts Medical Law and Ethics<br>Jan Gehrmann: Bachelor of Arts Sociology
3. Occupation	VH, JB, SM: research associates<br>JG: research assistant<br>ES: head of department, leader of research group, principal investigator<br>CB: head of department, principal investigator<br>JS, CBo: head of chair, principal investigator
4. Gender	VH, SM, ES, CB, CBo: Female<br>JB, JS: Male
5. Experience and training	Interviewers and researchers responsible for analysis had some to significant experience in qualitative research and were supervised and led by an experienced qualitative researcher (ES). Additionally, they attended external workshops and informal trainings at the department.<br><br>Experience<br>VH: University degree in medical ethics; significant experience in qualitative research and interviews: realized qualitative interview and focus group studies<br>JB: University degree in sociology; some experience in qualitative research: university courses<br>SM: University degree in Public Health; some experience in qualitative research: conducted qualitative interviews and focus groups previously<br>JG: University degree in sociology; some experience in qualitative research: university courses, conducted focus groups<br>ES: conceptualized and realized several qualitative interview studies previously
Relationship with participants	
6. Relationship established	Some participants were recruited from organizations affiliated with the researchers. Generally, participants were contacted by the researchers a few weeks before the interviews and had the

For Peer Review

	opportunity to establish a relationship. This was not possible in some cases, where interviewees were recruited "on the fly" (because the originally considered interviewees fell ill).
7. Participant knowledge of the interviewer	Interviewees were informed about the project, interviewer's educational background and occupational status in advance. Participants had the chance to request further information regarding the provided information.
8. Interviewer Characteristics	The interviewers have a research interest in health care research, especially in use of sedating drugs and sedation at the end of life. SM and ES were concurrently involved in a mixed-methods research project on sedation at the end of life in general palliative care.
**Domain 2: Study design**	
Theoretical Framework	
9. Methodological orientation and theory	Reported in the methods section "Design" (p1).
Participant selection	
10. Sampling	Reported in the methods section "Sampling" (p2).
11. Method of approach	Reported in the method section "Recruitment" (p1-2).<br><br>Additional information: Researchers contacted the contact persons at each research site, who then internally disseminated knowledge about the study and (pre-)recruited interviewees.
12. Sample size	Reported in the first paragraph of the results section (p3): 59 interviews
13. Non-participation	Reported in the discussion section "Strengths & Limitations" (p11): "During recruitment, the contact persons at the recruitment sites may have also inadvertently acted as gatekeepers by suggesting certain interview partners from the specialist palliative care teams without any clear guidelines or reasons for this pre-selection."<br><br>Additional information: As we did not receive any information on reasons for non-participation, some perspectives may not be fully taken into account.
Setting	
14. Setting of data collection	Reported in the methods section "Data collection" (p2-3): professionals' workplaces
15. Presence of non-participants	No one else was present besides the participants and the interviewer.
16. Description of Sample	Reported in the first paragraph of the results section (p3-4, Table 1).
Data Collection	
17. Interview Guideline	The development of the interview guide is briefly described in the methods section "Data collection" (p2-3).

For Peer Review

	Additional information:<br>The interview guide was pilot tested in six interviews. Due to only minor changes in the guide, those interviews were also included in analyses. Detailed versions of the interview guides can be requested from the authors (available in German language).<br>When JB joined the project, JB and VH conducted two interviews together to train JB. These were not included in the analysis.
18. Repeat interviews	No repeat interview was necessary.
19. Audio/Visual recording	Reported in the methods section “Data collection (p2-3).
20. Field notes	Reported in the methods section “Data collection” (p3): “During this time, field notes were written and VH and JB discussed preliminary impressions and findings with ES and other qualitative researchers, both from within and outside of the consortium”<br><br>Additional information: The field note form covered the following topics: relationship between researcher and participants, setting, account on interruptions, atmosphere, perceived moods or emotions of participants, difficulties in carrying out the interview, comments on content, feelings of the researcher. In case of a distinctive behaviour or strong emphasizing on certain topics, respective memos were added to the transcript.
21. Duration	Interview duration ranged between 40 and 110 minutes.
22. Data saturation	Reported in the methods section “Data collection” (p3): “Interviews were conducted until data saturation was achieved. “<br><br>Additional information: At the end of the indexing process, we confirmed saturation to be reached as no new themes emerged from the interviews.
23. Transcripts returned	Transcripts could not be returned to participants due to anonymization, which was demanded by our data protection officer. However, interviewers continuously mirrored and confirmed descriptions during the interview to guarantee correct understanding.
**Domain 3: Analysis and findings**	
**Data analysis**	
24. Number of data coders	Reported in the methods section “Data analysis” (p3):<br>Initial construction of the analytical framework: VH and JB, with support of ES<br>Indexing all interviews and refining the analytical framework: VH, JB, SM, and JG<br>Summarizing and charting of the indexed data: JB, SM, JG
25. Description of the coding tree	Reported in the methods section “Data analysis” (p3)

For Peer Review

	Additional information: To ensure quality and consistency of indexing/coding, a coding guide with detailed descriptions was developed by the coders. Analytical framework and coding guide are available from the authors upon request (in German language). Two researchers (VH, JB) independently indexed 10 transcripts and summarized a subset of the indexed data. Disagreements were discussed, partly involving a third researcher (ES), until consensus was reached. Additionally, we indexed and discussed part of the interview material together with our PPI group.
26. Derivation of themes	Reported in the methods section "Data analysis" (p3).
27. Software	Reported in the methods section "Data analysis" (p3): MAXQDA version 2018.2
28. Participant checking	We presented our results on a final conference, where healthcare professionals, including participants, could provide feedback.
Reporting	
29. Quotations presented	Quotations from different participants are presented to illustrate the findings, and a name (no relation to the real name of the participant possible) identifies each quotation.
30. Data and findings consistent	Yes
31. Clarity of major themes	Specialist palliative care professionals' views on dying under sedation emerged as an important aspect in the context of the broader research interest "sedation in specialist palliative care". We presented the major themes regarding professionals' perception of dying under sedation and discuss how it is related to what constitutes a good death.
32. Clarity of minor themes	As far as the word count permits, we discuss minor themes, too (for instance the image of the patient being peacefully at sleep).

For Peer Review
